# Anesthesia by Intra-Alveolar Injection

**Published:** 1908-10-15

**Authors:** F. L. Fossume


### ANESTHESIA BY INTRA-ALVEOLAR INJECTION.

BY F. L. FOSSUME, D.D.S.

It gives me great pleasure to appear before you this evening and offer a few words upon the comparatively new subject of anesthesia by intra-alveolar injection.

The greatest obstacle to the practice of dental surgery is the difficulty in anesthetizing the sensitive tissues of the teeth. Especially is this true with many who really need dental treatment most, as neurasthenic pregnant women and sensitive or nervous children. As a result high standards have had to be abandoned, and inferior and temporary operations performed, often resulting in the loss of permanent teeth by extraction. This last should be an unheard-of operation in modern dental surgery.

Many methods have been evolved for relieving the pain of dental surgery, but we must all admit that there is still much to be desired, and that any refinement in technique which contributes to that end is a boon to the patient and to the operator. Most of those present saw the demonstration of intra-alveolar injection at my clinic this afternoon. In the hands of others, as well as myself, this method of anesthetizing has given very excellent results, always greatly diminishing the sensibility and usually producing absolute anesthesia for all dental operations.

Dr. Kjennerud, of Norway, first aroused my interest in this method about eighteen months ago. He claimed to have a new formula for a local anesthetic. Being skeptical upon that subject, I inquired as to his technique, and was told that he used a very strong needle and forced it deeply into the alveolar process. Some experiments by Dr. H. S. Vaughan and myself showed us that when the needle penetrated the process deeply enough, an unusual amount of dental anesthesia was secured. There was however, so much bending and breaking of needles in attempting to force them through the dense outer plates of the alveolar process as to make the operation impractical. We found that any good sterile local anesthetic produced more or less complete anesthesia when forced into the cancelleous tissue, so that it reached the nerve supply at the apex of the root of the tooth.

Later Dr. Ottesen, also from Norway, told me to use a Beutel Rock drill for penetrating the alveolar process. The technique is as follows:

Every precaution must be taken to obtain absolute asepsis. The gums are washed with boric acid or bichloride solution, followed by alcohol. Touch the gum with phenol, and then inject one or two drops of the anesthetic intradermically. Trephine the alveolar plate with a No. 3 Beutel Rock drill. Be certain that the anesthetic is sterile. Use a No. 17 canula on the syringe and inject two to eight drops. The amount needed varies with the thickness and cancellation of the alveolar process. The perforation is always distal to the tooth, and usually a buccal injection is sufficient. Great care must be exercised in determining the length and direction of the molar roots. The injection should be made as close to the root apex as possible, otherwise partial anesthesia only may be obtained.

I am sure that all of us have experienced severe mental and nerve strain when performing radical operations upon hypersensitive tissues, such as the preparation of cavities with margins extended to the region of immunity, and proper step formation for the seating and anchorage of fillings and inlays. The extirpation of hyperemic pulps is very difficult with the usual anesthetic measures, and in many cases pulp stumps are permitted to remain. Sensitive dentine often prevents the correct preparation of abutments for bridge work. In many cases of pyorrhoea alveolaris the hyperesthesia of the tissues makes the curetting of abscess pockets and the thorough scaling and planing of the roots almost impossible.

I am daily convinced of the inestimable value of the intra-alveolar method of producing anesthesia. The above mentioned operations have been usually entirely free from pain. Patients have been quiet and unconscious of the nature of the work being done. Consequently there has been a great saving of time and energy. Therefore I have no hesitancy in urging you to give this method earnest consideration.—*Brief.* (Great care to use every antiseptic precaution, and only sufficient force to cause slow infiltration, should be made in any such method.—Editor DENTAL REGISTER.)

